# Determination and Dissection of DNA-Binding Specificity for the *Thermus thermophilus* HB8 Transcriptional Regulator TTHB099

**DOI:** 10.3390/ijms21217929

**Published:** 2020-10-26

**Authors:** Kristi Moncja, Michael W. Van Dyke

**Affiliations:** Department of Chemistry and Biochemistry, Kennesaw State University, Kennesaw, GA 30144, USA; kmoncja@students.kennesaw.edu

**Keywords:** bioinformatics, biolayer interferometry (BLI), electrophoretic mobility shift assay (EMSA), extremophile, protein-DNA binding, type IIS restriction endonuclease

## Abstract

Transcription factors (TFs) have been extensively researched in certain well-studied organisms, but far less so in others. Following the whole-genome sequencing of a new organism, TFs are typically identified through their homology with related proteins in other organisms. However, recent findings demonstrate that structurally similar TFs from distantly related bacteria are not usually evolutionary orthologs. Here we explore TTHB099, a cAMP receptor protein (CRP)-family TF from the extremophile *Thermus thermophilus* HB8. Using the in vitro iterative selection method Restriction Endonuclease Protection, Selection and Amplification (REPSA), we identified the preferred DNA-binding motif for TTHB099, 5′–TGT(A/g)NBSYRSVN(T/c)ACA–3′, and mapped potential binding sites and regulated genes within the *T. thermophilus* HB8 genome. Comparisons with expression profile data in TTHB099-deficient and wild type strains suggested that, unlike *E. coli* CRP (CRP_Ec_), TTHB099 does not have a simple regulatory mechanism. However, we hypothesize that TTHB099 can be a dual-regulator similar to CRP_Ec_.

## 1. Introduction

Transcription factors (TFs) are DNA-binding proteins that allow for modulation of transcription initiation in response to intracellular and extracellular changes. Over decades of research, there have been many advances in exploring the TFs regulatory mechanisms cells use to control their gene expression. However, technological innovations such as massively parallel sequencing and data sciences have expanded our interest in new model organisms and their adaptations. TFs are *trans* factors that bind to *cis*-regulatory elements, promoter or enhancer sequences known as TF binding sites (TFBSs). It has been reported that most of the bacterial TFBSs are found in the proximal region (about −100 to +20 bp from the transcription start site [TSS]) and distal regions (up to −200 from TSS) [1,2,3]. Functionally, TFs are categorized into activators and suppressors, with a few of them being dual-regulators [4]. Regarding the number of genes regulated, TFs are classified into local or global regulators [5]. Such characteristics make up the mechanism of transcription regulation and help identify novel TFs.

Proteomic studies allow the grouping of TFs into families based on structural comparison studies. However, new findings have shown that structurally similar TFs from distantly related bacteria are not usually evolutionary orthologs [6]. A more comprehensive characterization of the TF regulatory network is achieved by identifying the TFBSs, the genes regulated, and the method of regulation. Advances in computational biology and data processing have given rise to inclusive databases that can predict structure and function for TFs in new model organisms [7]. However, most of these databases are built from experimental studies. 

To gain insights into transcriptional regulatory networks in extremophile organisms, our laboratory has employed a novel biochemistry-based method, Restriction Endonuclease, Selection, Protection, and Amplification (REPSA), to characterize several TFs in the extreme thermophilic model organism *Thermus thermophilus* HB8. To date, we have studied four tetracycline repressor protein (TetR) family transcriptional suppressors and have successfully identified their TFBSs [8,9,10,11]. Commonly, suppressors bind DNA in the absence of small-molecule modulators/cofactors and with high-affinity. Contrary, numerous transcriptional activators employ small-molecule modulators in order to bind DNA, thus complicating their analysis in vitro. 

In this study, we explore the utility of REPSA to identify and characterize a potential thermophilic transcriptional activator, TTHB099. Protein sequence homology analysis indicates that TTHB099 is one of the four cAMP receptor protein (CRP) family members (TTHA1437, TTHA1359, TTHB099, and TTHA1567) in *T. thermophilus* HB8 and should bind palindromic DNA sequences as a homodimer [12]. However, despite having a cAMP binding domain, it does not require this cofactor to bind DNA. Here, we identified the preferred DNA-binding sequence for TTHB099 as the 16-mer motif: 5′–TGT(A/g)n(t/c)c(t/c)(a/g)g(a/g)n(T/c)ACA–3′. Furthermore, we used binding kinetics studies and mRNA expression data to validate potential biological roles of TTHB099. 

## 2. Results

### 2.1. Preferred TTHB099-Binding Sequences Selected Via REPSA

**Figure 1:**
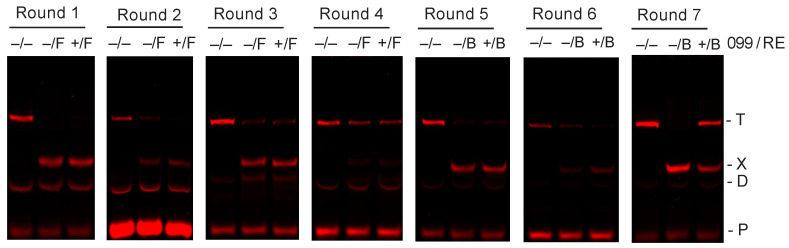
Selection of TTHB099-binding DNA sequences. Shown are IR fluorescence images of restriction endonuclease cleavage-protection assays made during Rounds 1–7 of REPSA selection with 50.6 nM TTHB099 protein. The presence (+) or absence (−) of TTHB099 and IISRE FokI (F) or BpmI (B) are indicated above each lane. The electrophoretic mobility of the intact (T) and cleaved (X) ST2R24 selection template, primer dimer species (D), as well as the IRD7_ST2R primer (P) are indicated at the right of the figure.

REPSA was used to select for TTHB099-binding sites present in a large pool (~60 billion molecules) of synthesized double-stranded DNA. Our selection library, ST2R24, has been successfully used in four previous studies [8,9,10,11]. IRDye^®^ 700 (IRD7)-labeled library DNA was incubated with purified TTHB099 protein to permit specific binding, then challenged by a type IIS restriction endonuclease (IISRE). Sequence-specific binding of TTHB099 to a subset of the library protected those oligonucleotides from endonuclease activity, thereby permitting their amplification by PCR. Seven rounds of binding, IISRE cleavage, and PCR resulted in the enrichment of DNA resistant to IISRE cleavage when TTHB099 was present (Figure 1, Round 7). Note that in Round 4, substantial uncut DNA appeared on the IISRE control lane (–/F) as well as the test lane (+/F). This nonspecific cleavage inhibition has been observed before and has been ascribed to the selection of FokI cleavage-resistant sequences [8,13]. Thus, subsequent rounds of REPSA were performed with an alternative, albeit less efficient IISRE (BpmI).

**Figure 2:**
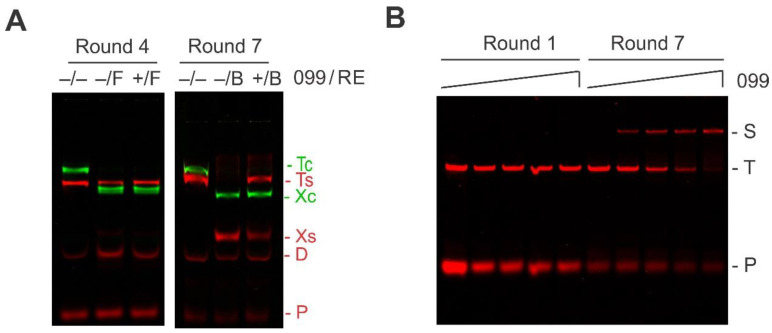
Validation of TTHB099–binding DNA sequences. (**A**) Shown are IR fluorescence images of restriction endonuclease protection assays made with DNA from Round 4 and 7 of REPSA selection. The presence (+) or absence (−) of TTHB099 and IISRE FokI (F) or BpmI (**B**) are indicated above each lane. The electrophoretic mobility of the intact (T) and cleaved (X) IRD8-labeled REPSAis control DNA (green, T_c_ and X_c_), IRD7-labeled ST2R24 selection template (red, T_s_ and X_s_), primer-dimers (D), as well as the IRD7_ST2R primer (P) are indicated at the right of the figure and color-coded to match the fluorescently labeled DNA present. (B) Shown are IR fluorescence images of electrophoretic mobility shift assays made with DNA mixtures obtained from Round 1 (left lanes) and Round 7 (right lanes) of REPSA selection incubated with increasing concentrations of TTHB099 protein (from left to right: 0, 5.06, 50.6, 506, and 5060 nM TTHB099). The electrophoretic mobility of a single protein-DNA complex (S) as well as the uncomplexed ST2R24 selection template (T) and IRD7_ST2R primer (P) are indicated at the right of the figure.

Before proceeding, it is prudent to validate our selection of TTHB099-binding sequences. To do so, REPSA-selected DNA was subjected to a restriction endonuclease protection assay (REPA), which is very much like the binding and IISRE cleavage steps of REPSA [14]. The inclusion of a different fluorophore-labeled control DNA in these reactions permitted the discrimination of TTHB099-specific and nonspecific IISRE cleavage inhibition. Thus, Round 7 DNA exhibited the expected pattern of cleavage protection expected for a majority population of TTHB099-binding DNA (Figure 2A). However, Round 4 DNA exhibited TTHB099-independent cleavage protection of the selected DNA, consistent with a majority being refractory to cleavage by the IISRE FokI. Additional validation was achieved using an electrophoretic mobility shift assay (EMSA) to directly visualize TTHB099-DNA complexes. In this independent assay, different concentrations of TTHB099 protein were incubated with Round 1 and Round 7 DNA prior to electrophoresis (Figure 2B). The slower mobility of the DNA-protein complex present in Round 7 but not in Round 1 DNA indicated that a substantial portion of the selected sequences contained stable TTHB099 binding sites. The results from REPSA and EMSA encouraged further studies on determining TTHB099-DNA binding sequences.

### 2.2. Identification and Characterization of TTHB099-Binding Motif

**Figure 3:**
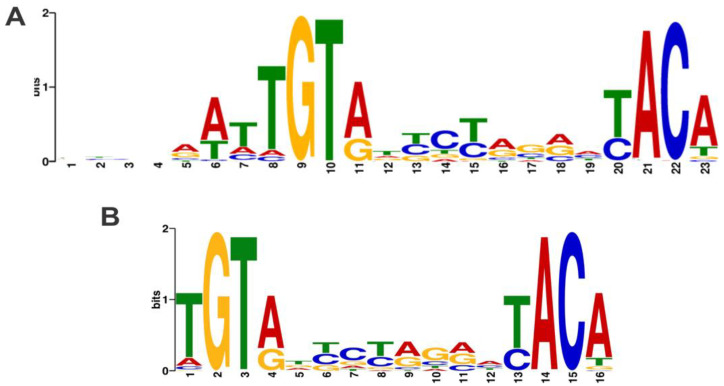
TTHB099-binding motifs. Sequence logos were determined using MEME software with an input of 1000 Round 7 DNA sequences. (**A**) MEME performed with no filters. (**B**) MEME performed using a palindromic filter.

To massively parallel sequence the REPSA-selected DNA, Round 7 DNA was amplified with fusion PCR primers, purified, and emulsion PCR amplified onto individual sequencing particles (ISPs). The enriched ISPs were subjected to next-generation semiconductor sequencing using an Ion Personal Genome Machine (PGM) system. The multiplexed sequencing run yielded 6,921,164 total bases, 6,169,384 ≥ Q20, resulting in 120,585 reads of 57-bp mean length for the TTHB099 Round 7 DNA. A randomly selected set of 1000 reads was input into web version 5.0.5 of Multiple Em for Motif Elicitation (MEME) analyzed using default parameters with and without a palindromic filter [15]. The output position weight matrices displayed the best 23-mer motif without filters with an E-value of 2.4 × 10^−2234^ (Figure 3A), and the best 16-mer palindromic motif with an E-value of 2.4 × 10^−2871^ (Figure 3B). These statistically significant results indicate that the identified motifs are likely consensus sequences for the TTHB099 transcription factor.

**Figure 4:**
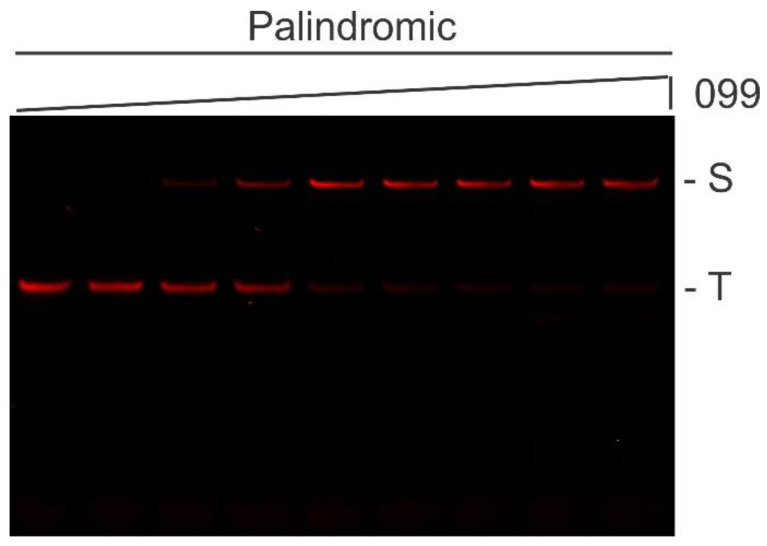
EMSA analysis of TTHB099 binding to its palindromic consensus sequence. Shown is an IR fluorescence image of IRD7-labeled ST2_099 incubated with 0, 0.66, 1.32, 2.64, 5.27, 10.5, 21.1, or 42.2 nM TTHB099 protein. (S) Protein-DNA complex, and (T) uncomplexed DNA.

Noting that the nonpalindromic sequence logo is an extended version of the palindromic one, with seven vaguely significant nucleotides upstream, it was postulated that the palindromic logo is a better representation of the TTHB099 consensus DNA-binding sequence. To test this hypothesis, the 16-mer sequence 5′–TGTATTCTAGAATACA–3′ was incorporated into an ST2 background, yielding the probe ST2_099. A fixed concentration of IRD7-labeled ST2_099 was incubated with increasing purified TTHB099 protein concentrations to permit specific binding and the resulting products analyzed by EMSA (Figure 4). We found that the TTHB099-ST2_099 complex exhibited similar electrophoretic mobility as observed with the TTHB099-Round 7 DNA complex (Figure 2B), suggesting that most Round 7 DNA contained the palindromic sequence. Quantitative densitometry analysis of the fourth lane gives an approximate dissociation constant (K_D_) of 4.5 nM (Supplementary Figure S1, Supplementary Table S1).

**Figure 5:**
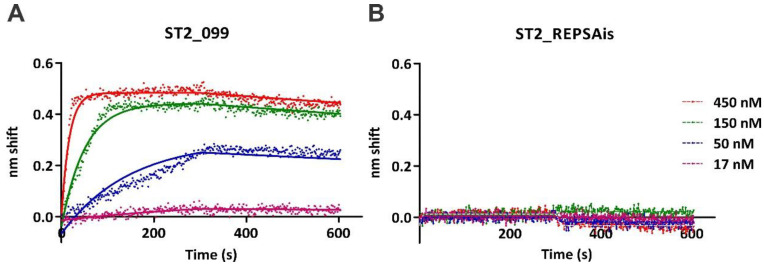
Biolayer interferometry analysis of TTHB099 binding to DNA. Shown are raw traces (dots) and best-fit lines of TTHB099 binding to (**A**) ST2_099 consensus DNA and (**B**) ST2_REPSAis control DNA TTHB099. Concentrations investigated include 450 nM (red), 150 nM (green), 50 nM (blue), and 17 nM (magenta).

Biolayer interferometry (BLI) was used to characterize TTHB099-consensus DNA interactions. This innovative approach measures in vitro real-time interactions between macromolecules, including proteins and nucleic acids [16]. Our BLI analysis involved biotinylated consensus sequence, ST2_099, affixed onto streptavidin sensors interacting with increasing TTHB099 protein concentrations in solution. This provided a qualitative observation of protein-DNA association and dissociation kinetics (Figure 5A). The most substantial interactions were observed for the highest concentrations of TTHB099 (450 nM (red) and 150 nM (green)). An arbitrary DNA sequence, ST2_REPSAis, was tested as a control DNA (Figure 5B). It demonstrated binding interactions that were below our experimental detection levels, consistent with a low TTHB099-REPSAis affinity. Another outcome of this study was the quantitative evaluation of the TTHB099-consensus binding affinity. Least squares regression analysis of the association and dissociation rates were calculated with GraphPad Prism 8. From those rates, a dissociation constant was produced. TTHB099 interacting with its consensus sequence had a K_D_ of 2.214 nM with an R^2^ value of 0.9883.

Further characterization of TTHB099-DNA binding was made using selected point mutations of its consensus sequence and BLI. Binding kinetics data, including association rate (k_on_), dissociation rate (k_off_), and the dissociation constant, were derived for each of the mutated sequences and displayed in Table 1. As observed with the m2 mutant, a single change in a highly conserved nucleotide of the consensus sequence affects the binding affinity by 15-fold. Even point mutations of less conserved positions (e.g., m5) decreased affinity by 2-fold. These data suggest that the TTHB099 binding to DNA is highly sequence-specific. Additionally, the nanomolar dissociation constant we observed indicates that our consensus sequence is a good representation of the native TTHB099’s preferred sequences in *T. thermophilus* HB8. Notably, TTHB099-DNA binding is not affected by the absence or presence of the second messenger 3′,5′ cAMP, unlike its archetype protein CRP_Ec_ [17].

### 2.3. T. thermophilus HB8 Genome-Wide Mapping of the TTHB099-Binding Motif

The Find Individual Motif Occurrences (FIMO) program was used to scan the *T. thermophilus* HB8 genome (GenBank uid13202 210) for the 16-mer palindromic sequence identified through MEME software [18]. FIMO revealed 78 motif occurrences with a *p*-value of less than 0.0001. The top 25 results with *p*-values ≤ 3.95 × 10^−5^ are shown in Table 2. The locations of these 25 sequences relative to the transcription start site of their proximally downstream genes were determined using the Kyoto Encyclopedia of Genes and Genomes (KEGG) and verified in the National Center for Biotechnology Information (NCBI) database [19,20]. Furthermore, operon predictions for each location were made using the Database of PrOkaryotic OpeRons (DOOR^2^) and BioCyc [21,22]. Sixteen of these sites were situated within the −200 to +20 nucleotide region most common for transcription activator binding. Furthermore, their proximally downstream genes were the first of their operons or single transcriptional units, making these sites stronger candidates for TF regulation. The other nine sites were omitted from further analysis because they were located further downstream, inside open reading frames, or, as in the case of *TTHC003*, too far upstream (−666 nucleotides).

**Figure 6:**
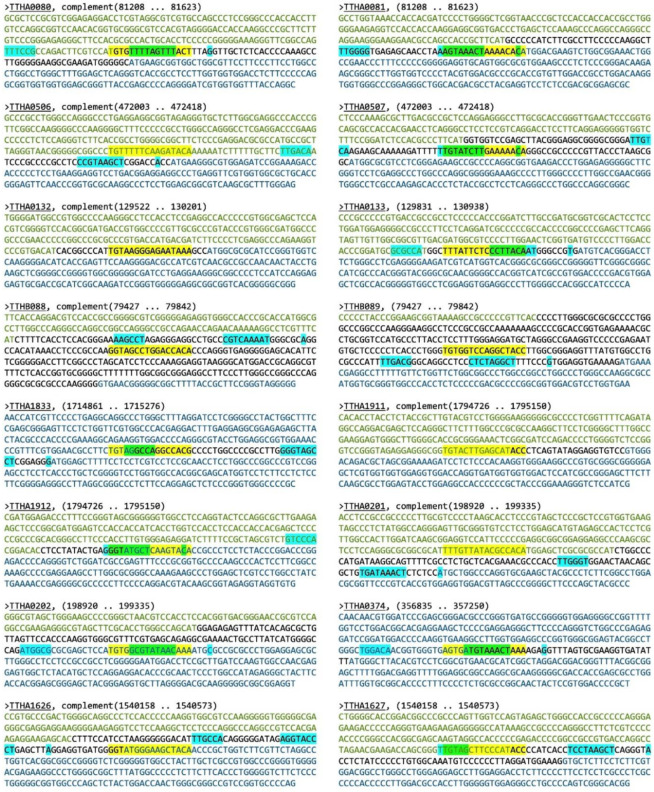
Promoter predictions of sequences potentially regulated by TTHB099 within the *T. thermophilus* HB8 genome. Shown are ±200 bp sequences from the TTHB099 binding site identified through FIMO (see Table 2). Blue nucleotides represent the longest open reading frames with a downstream orientation relative to the TTHB099 binding site; Green nucleotides indicate open reading frames with the opposite orientation; Black nucleotides imply intergenic regions. Potential promoter elements (−35 and −10 boxes, +1 start site of transcription) are indicated with cyan highlighting; TTHB099-binding sites are indicated with yellow highlighting; Overlapping TTHB099-binding and core promoter elements are indicated by green highlighting.

To better ascertain a potential role for TTHB099 to regulate transcription, all the 16 sequences selected from FIMO were analyzed for potential core promoter elements. Sequences ± 200 bp upstream and downstream of the FIMO identified TTHB099-binding sites were evaluated in SoftBerry BPROM (Figure 6) [23]. Many sequences (9/16) contained a TTHB099-consensus sequence that overlapped with at least one promoter element (−35 box, −10 box, +1 start site). Those included *TTHA0081/80, TTHA0507, TTHA0133, TTHA1833, TTHA1912, TTHA0202, TTHA0374*, and *TTHA1627*. Three of the TTHB099-binding sequences, *TTHA0506, TTHB089*, and *TTHA0201,* were located upstream of the nearby −35 box. Conversely, *TTHB088* and *TTHA1626* had their putative TTHB099-binding sequences located downstream of the postulated promoter elements. There were no identified promoter elements near *TTHA0132* and *TTHA1911.* It is not clear why BPROM was unable to identify any core promoter elements for these genes, but limitations could arise from a potential difference between core promoter elements in *E. coli*, the model organism used by BPROM, and those of *T. thermophilus* HB8.

### 2.4. Validation of Potential TTHB099-Regulated Genes

Apart from analyzing the locations of the binding sequences concerning the TSS, as well as their positions regarding promoters, we investigated the affinity of TTHB099 protein for the selected sequences. To better understand how TTHB099 regulates genes identified through FIMO, all 16 sequences underwent binding kinetics analysis using BLI. As some TTHB099 binding sites are shared by two bidirectional promoters, only nine unique sequences were synthesized into biotinylated double-stranded oligonucleotides. Binding reactions containing four different concentrations of TTHB099 (450, 150, 50, and 17 nM) were tested against each binding site probe (Table 3). The strongest binding was observed for *TTHA1833* and *TTHB088/89*, with K_D_ values below 10 nM. The genes with binding affinities between 10–100 nM were *TTHA1911/12*, *TTHA0506/07*, and *TTHA0080/81* in increasing order. *TTHA1626/27*, *TTHA0132/33*, and *TTHA0201/02* displayed the weakest binding, with K_Ds_ > 100 nM, while binding to *TTHA0374* could not be detected under our experimental conditions. Interestingly, these binding parameters do not always follow the sequence order defined by FIMO, suggesting that there could be other factors at play that are not considered by this in vitro analysis.

Further validation of TTHB099 involvement in the transcriptional regulation of these genes was sought through the analysis of prior DNA microarray studies, publicly available through the National Center for Biotechnology Information Gene Expression Omnibus (NCBI GEO) [24]. A GEO2R comparison (SuperSeries GSE21875, Supplementary Table S2) of expression profile data from sets of TTHB099-deficient and wild type strains was used to determine if the absence of TTHB099 produced any substantial changes in the expression of the FIMO-identified genes. Of these genes, only *TTHA1626* displayed a substantially increased expression with a logFC of 2.62. The remainder of the 15 genes had only small, non-significant changes, as shown in Table 4. Likewise, individual genes within their respective operons did not seem to have any significant changes.

As an additional approach to better understand potential gene regulation by TTHB099, we investigated the postulated biological functions of these genes. Many were reported only as encoding hypothetical proteins, which is fairly common in *T. thermophilus*. Several encoded proteins that may be involved in sugar metabolism (malate synthase, 3-isopropylmalate dehydratase), energy metabolism (3-isopropylmalate dehydratase large subunit, homoaconitate hydratase small subunit), or transport. Most interesting, two genes (*TTHA0134* and *TTHA0507*) are believed to encode transcriptional regulators. If so, their expression could complicate the identification of directly TTHB099-regulated genes by GEO2R.

Another analysis of the GEO2R data was focused on investigating the genes that were affected most by the absence of TTHB099 (Table 5). These genes could be grouped into operons, suggesting that their expression was not affected by multiple-unrelated TFs, but rather a fundamental regulatory mechanism involving TTHB099. The upregulated genes, 75% (50/67), were involved in the electron transport chain (ETC) of oxidative phosphorylation, carbohydrate metabolism, bacteria motility, and osmotic stress defense. The downregulated operons, 25% (17/67 genes), were related to ribosomal proteins, ion ABC transporters, and ATPases. MEME analysis of the −300/+100 bp sequences upstream of each operon did not find our TTHB099 consensus sequence or reveal any additional binding motifs. Taken together, this suggests a complicated mechanism for the regulation of these genes that may not involve TTHB099 directly regulating their transcription.

## 3. Discussion

In this study, an in vitro iterative selection method, REPSA, was used to annotate the TTHB099 transcription regulator in *T. thermophilus* HB8. This, coupled with next generation sequencing and MEME motif elicitation, allowed for the identification of the TTHB099-DNA binding motif, a 16 bp long palindromic sequence, 5′–TGT(A/g)n(t/c)c(t/c)(a/g)g(a/g)n(T/c)ACA–3′, with a consensus half-site 5′–T_1_G_2_T_3_(A/G)_4_N_5_(T/C)_6_C_7_(T/C)_8_–3′. Binding kinetics between TTHB099 and its consensus sequence, as well as single point mutations within its half-site, were investigated using BLI. TTHB099 protein bound the 16-mer consensus sequence with a high affinity (K_D_ = 2.21 nM) and the point-mutated sequences in the range of 4.86 of 33.6 nM with mutations at the second and third positions having the greatest effect. The different binding affinities for each mutated sequence mirrored the MEME results represented by the TTHB099 sequence logo. Our report is the first time a consensus sequence has been identified for TTHB099.

Interestingly, our sequence has a strong resemblance to the *E. coli* CRP (CRP_Ec_) consensus sequence, 5′-AAATGTGATCTAGATCACATTT-3′ [26]. In both cases, the trimers “TGT” and “ACA” are highly conserved and are considered most significant for TF binding. The specifics of this resemblance could be correlated to the homology between the two proteins previously reported by Agari et al. [12]. However, *E. coli* and *T. thermophilus* HB8 are not only phylogenetically distant, but they also live in entirely different environments, mesophilic and extremophilic, respectively [27]. Hence, the biological roles of TTHB099 need not necessarily be the same as those of CRP_Ec_. This is most evident in the observation that TTHB099 does not require the second messenger 3′,5′ cAMP to bind DNA, which is required by CRP_Ec_.

Having found and validated a consensus TTHB099-binding sequence, mapping it onto the genome of *T. thermophilus* HB8 would help identify potential TTHB099-regulated genes. Using FIMO, the MEME derived position weight matrix version of our consensus sequence recognized 78 sequences. The top 25 sequences with the best *p*-values were selected for further validation. It is important to note that the *p*-values derived were not as small as found in our previous studies, due to the ten poorly conserved positions in the middle of the TTHB099 consensus sequence palindrome, which affected the dynamic programming algorithm of FIMO. Our analysis of the TTHB099 binding site location relative to the TSS of the proximal downstream genes showed that almost half of the identified sites were located inside open reading frames, which is not typical for traditional transcription factors. Notably, no potential TTHB099 binding site was found near its own gene. This could imply that the TTHB099 TF by itself has no direct regulatory role over its operon litR (*TTHB100, TTHB099, TTHB098*) or the divergent crtB operon (*TTHB101, TTHB102*) that shares a common intergenic region. Autoregulation is a common feature for many prokaryotic TFs, including members of the CRP family, but may not be a characteristic for TTHB099 unless in an auxiliary fashion [28].

The promoter analysis revealed that nine TTHB099-binding sites overlapped with potential core promoter elements, a TF-promoter interaction characteristic of Class II transcription activators, as well as transcription inhibition via steric hindrance. Additionally, three sequences are located upstream of the −35 box, fitting the Class I activator model, while two are downstream of the −10 box, a model used by both transcription activators and repressors. These variations in the binding method suggest that TTHB099 could be either an activator or a suppressor. Indeed, the dual regulatory role is common in global regulators such as CRP_Ec_ [29]. Moreover, eight pairs of the TTHB099-binding sequences were found in the intergenic region of divergent genes, another characteristic of dual-regulators [30].

Biophysical studies performed with BLI were used to further our understanding of TTHB099 interaction with the identified sites. The equilibrium dissociation constants were below the micromolar range, showing that TTHB099 had some appreciable affinity for the tested sites. However, variations as high as 200-fold were observed. These K_D_ changes did not follow any particular trends, such as the *p*-value order established through FIMO. Neither did the sites with the highest affinity have similarities in terms of promoter location or presumed manner of transcription regulation. For example, the TTHB099 binding sequence with the highest affinity (3.05 nM) was located in the intergenic region and overlapped with the −35 box upstream of *TTHA1833*. The TTHB099 binding sequences with the next lowest K_D_ were also situated in the intergenic regions, but they were located upstream and downstream of the *TTHB088/89* promoters, respectively. Such biophysical results emphasize the importance of experimental validation of theoretically determined sites.

Our BLI binding studies are limited to the simple interactions of purified protein with synthesized DNAs in the absence of any environmental or biological factors. Knowing that the transcription regulation apparatus can be complex, we decided to complement our in vitro study with data from in vivo expression profiles. Using publicly available expression profile data from the matched wild type and TTHB099-deficient *T. thermophilus* HB8 strains, operons of the 16 potentially regulated genes were investigated. We found that the mRNAs of these genes were not significantly affected by the deficiency of TTHB099. These results suggest that TTHB099 does not have, on its own, any appreciable regulatory roles over these genes in exponentially propagating wild type organisms. 

Nonetheless, TTHB099 deficiency does appreciably affect the expression of several genes in exponentially propagating *T. thermophilus* HB8. We identified 19 operons, 12 of which were overexpressed (positively affected) in the deficient strains. The upregulated set of genes were involved in the electron transport chain (ETC) of oxidative phosphorylation, sugar metabolism, type IV pilin related proteins, and one osmotically inducible protein, consistent with TTHB099 being a transcriptional repressor. Conversely, there were seven under-expressed operons or a total of 17 genes in the TTHB099-deficient strains, suggesting that TTHB099 may act as an activator for these genes. The downregulated genes encoded for ribosomal proteins, iron ABC transporters, and ATPases. Notably, the biological roles of the most affected operons in the TTHB099-deficient strain were involved in metabolic pathways that have been reported to be regulated by the archetype CRP_Ec_ [31]. For example, ribosome related genes were downregulated in the absence of *TTHB099*, similar to what Pal et al. reported for their evolutionary expressed CRP_Ec_-deficient strains [32]. Likewise, iron transport genes were downregulated in the absence of *TTHB099*, similar to what was observed in the absence of CRP_Ec_, as Zhang et al. reported [33]. Such results indicate that TTHB099 does have some biological functions similar to those of the CRP_Ec_. However, these regulatory roles do not seem to be affected by changes in cAMP concentration. Moreover, a MEME search for a consensus sequence between the 19 most-affected operons identified via the GEO data failed to bring up any significant motifs. Thus, the hypothesis for a simple regulatory mechanism is once more unsatisfied. 

TT_P0055 from *T. thermophilus* HB27, an ortholog of TTHB099 with only one amino acid substitution (E77D), has been reported to be a positive regulator of *crtB* operon, which in turn is involved in light-dependent carotenoid biosynthesis [33]. However, the functional effects of TT_P0055 on carotenoid production lack details on the mechanism of regulation and could indicate that TT_P0055 has indirect control over *crtB* activation. The homology between the HB27 and HB8 strains, particularly on this regulatory complex (TT_P0055 and TTHB099 proteins, their intergenic regions, and their *crtB* operons), would suggest similar biological functions for the two TFs. When analyzing the GEO expression data in the absence of TTHB099, there is no detectable change in *crtB* genes. These results could be attributed to the absence of light in the experimental conditions required to deplete the litR transcriptional repressor of TT_P0055, the latter positively regulating carotenoid production [34].

Because TTHB099 does not seem to have any observable binding to the *crtB* promoter, the study published by Ebright et al. centered on TTHB099 binding upstream of *TTHB101* is based on a prediction not firmly established [35]. Hence, Ebright’s claim that TTHB099 is a model class II transcription activator may need to be reconsidered under the light of our new findings.

Looking for a connection between the genes found via the REPSA-identified consensus sequence and the genes affected by TTHB099 deficiency, as determined by GEO2R, we found that five of the affected operons (30 genes) had an upstream binding sequence identified by FIMO. Interestingly, these binding sites were located at about 0.9 to 4 kbp upstream of the most affected operons. Such behavior could be explained by TTHB099 acting as an enhancer or silencer. These elements do exist in the prokaryotic world but not in large numbers. To date, the identified prokaryotic enhancers regulate only a few promoters used by σ^54^-directed RNA polymerases [36]. Knowing that *T. thermophilus* HB8 does not have a σ^54^ homolog, it becomes even more challenging to suggest that TTHB099 can function as an enhancer/silencer. Future studies could be designed to analyze potential interactions of TTHB099 with other TFs, supporting the hypothesis of a complex regulatory mechanism involving distal enhancer/silencer elements. As for TTHB099 being an activator or a suppressor, all our data point towards a dual regulatory role.

## 4. Materials and Methods 

### 4.1. Preparation of Oligonucleotides

Single-stranded oligonucleotides used in this study (Supplementary Table S3) were obtained from Integrated DNA Technologies (Coralville, IA). ST2R24 library DNA used for the initial REPSA round was PCR amplified with primers ST2L and IRD7_ST2R for seven cycles to ensure maximal double-stranded DNA content with fully annealed randomized cassette regions. Subsequent REPSA round DNAs were PCR amplified for 6, 9, and 12 cycles to identify those products with optimal cassette integrity. Libraries for massively parallel semiconductor sequencing were prepared by a two-step fusion PCR process, using primers A_BC01_ST2R and trP1_ST2L as the initial set and A_uni and trP1_uni as the second set, as previously described [8]. Other duplex DNAs were prepared by conventional PCR amplification following the Taq DNA polymerase manufacturer’s instructions. EMSA probes were amplified with primers ST2L and IRD7_ST2R, while nucleic acids used in BLI assays were amplified with primers ST2L and Bio_ST2R. The concentrations for the modified oligonucleotides were measured with Qubit 3 Fluorometer following our protocol [37].

### 4.2. TTHB099 Protein Expression and Purification

TTHB099 protein was expressed following IPTG induction of *E. coli* BL21(DE3) bacteria transformed with plasmid PC014099-42 (obtained from RIKEN Bioresource Research Center) and purified from soluble bacterial extracts by heat-treatment as described in our previous study [11]. SDS-PAGE analysis of fractions from purification steps is shown in Supplementary Figure S2A and is consistent with a near quantitative recovery of TTHB099 protein. Analysis by quantitative densitometry with Coomassie Brilliant Blue staining indicated that the purified TTHB099 preparation had a final concentration of 50.6 μM (Supplementary Figure S2B). 

### 4.3. TTHB099-Consensus Sequence Determination

REPSA selections with 50.6 nM TTHB099 were performed essentially as previously described [8], with the exception that 3.2 U FokI were used in Rounds 1–4 and 8 U BpmI were used in Rounds 5–7. Furthermore, the Round 1 reactions were seeded with 4.515 ng (100 fmol) ST2R24 DNA pool. The PCR amplification reactions were adjusted to use 560 nM of primers and 25 U NEB *Taq* polymerase. Finally, the annealing and elongation temperatures were adjusted to 58 °C and 68 °C, respectively. 

The amplicon library preparation, Ion PGM individual sequencing particle (ISP) preparation, Ion PGM semiconductor sequencing, and Ion Torrent sever sequence processing were all performed as previously described [8]. Resulting raw sequences in fastq format (Supplementary Data S1) were further processed by our Sequencing1.java program [8] and DuplicatesFinder v 1.1 (http://proline.bic.nus.edu.sg/~asif/tools/DuplicateFinder.zip) to yield data (Supplementary Data S2) suitable for consensus sequence determination by web version 5.0.5 of Multiple Em for Motif Elicitation (MEME) (http://meme-suite.org/tools/meme) [15]. Position-weight matrices for the top three motifs were determined and displayed as sequence logos, from which a consensus sequence was derived.

### 4.4. Protein-DNA Binding Assays

Electrophoretic mobility shift assays (EMSA) with both libraries and defined DNA were performed as previously described [8], with a detailed protocol being available [38]. Note that EMSA experiments performed with REPSA selected DNAs contain multiple DNA species, including high concentrations of DNA primers, and should not be used to determine apparent binding affinities. Biolayer interferometry was performed as previously described [11], with the exception that only four concentrations of TTHB099 (17, 50, 150, 450 nM) were used for each DNA probe investigated. Such was sufficient to yield global values for *k*_on_ and *k*_off_ rate constants as well as K_D_ equilibrium binding constants with R^2^ goodness-of-fit determinations of greater than 0.95 in all cases. A single BLI experiment was performed with 2.25 nM consensus (wt) probe, 200 nM TTHB099, and 100 nM 3′,5′-cAMP, to test the effects of cAMP on TTHB099-DNA binding. Its R^2^ value was 0.92.

### 4.5. Bioinformatic Determination of Candidate Regulated Genes

The 16-bp position weight matrix obtained from a MEME analysis of our processed sequencing data was used as the input for FIMO analysis (http://meme-suite.org/tools/fimo) [18], to identify best matches within the T. thermophilus genome. Stringency was limited to include matches having *p*-values ≤ 3.95 × 10^−5^. Sequences ±200 bp from the TTHB099 binding site were analyzed by Softberry BPROM (http://www.softberry.com) [23] to identify potential bacterial core promoter elements. Operons were identified using the Database of PrOkaryotic OpeRons (DOOR^2^) (http://161.117.81.224/DOOR3/annotate.php) and BioCyc (http://biocyc.org) [21,22]. Putative biological functions of TTHB099-regulated genes were obtained using T. thermophilus HB8 data from KEGG (https://www.genome.jp/kegg-bin/show_organism?org=T00220) [19]. Publicly available microarray data for gene expression profiles in wild-type and TTHB099-deficient T. thermophilus HB8 were obtained from the NCBI GEO website (https://www.ncbi.nlm.nih.gov/geo/) SuperSeries GSE21875 [24]. In particular, samples GSM532194, 5, and 6, obtained from wild-type *T. thermophilus* HB8 grown in a rich medium for 360 min and samples GSM530118, 20, and 22, obtained from TTHB099-deficient *T. thermophilus* HB8 strains propagated under identical conditions. These data sets were analyzed using their NCBI GEO2R program with default settings to determine changes in gene expression (LogFC values) and their statistical significance (*p*-values).

## Figures and Tables

**Table 1:** TTHB099-DNA binding parameters for consensus and mutant sequences.

Name	Sequence	*k*_on_ (M^−1^s^−1^)	*k*_off_ (s^−1^)	K_D_ (M)	R^2^
wt	TGTATTCTAGAATACA	131,308	2.907 × 10^−4^	2.214 × 10^−9^	0.9883
m1	gGTATTCTAGAATACA	120,059	7.558 × 10^−4^	6.295 × 10^−9^	0.9895
m2	TtTATTCTAGAATACA	112,773	3.785 × 10^−3^	3.356 × 10^−8^	0.9778
m3	TGaATTCTAGAATACA	88,146	1.221 × 10^−3^	1.385 × 10^−8^	0.9824
m4	TGTcTTCTAGAATACA	142,953	1.366 × 10^−3^	9.557 × 10^−9^	0.9817
m5	TGTAcTCTAGAATACA	110,766	5.379 × 10^−4^	4.856 × 10^−9^	0.9879
m6	TGTATaCTAGAATACA	125,945	7.064 × 10^−4^	5.608 × 10^−9^	0.9794
m7	TGTATTtTAGAATACA	119,827	6.978 × 10^−4^	5.823 × 10^−9^	0.9805
m8	TGTATTCaAGAATACA	115,299	7.848 × 10^−4^	6.807 × 10^−9^	0.9840
wt + cAMP	TGTATTCTAGAATACA	214,759	4.780 × 10^−4^	2.226 × 10^−9^	0.9231

(Sequence) Lowercase nucleotides indicate a mutation from the TTHB099 consensus sequence (wt). (wt + cAMP) Binding reactions performed with the consensus sequence in the presence of 100 nM 3′,5′cAMP.

**Table 2:** TTHB099-consensus sequences mapped in the genome of *T. thermophilus* HB8.

Start	End	*p*-Value	*Q*-Value	Sequence	Loc	Gene	Op
81,408	81,423	4.03 × 10^−6^	1	AGTAAACTAAAACACA	+1	*TTHA0081*	1/3
81,408	81,423	4.03 × 10^−6^	1	TGTGTTTTAGTTTACT	−48	*TTHA0080*	S
32,704	32,719	5.82 × 10^−6^	1	TGTGTACGAAATTACA	+434	*TTHA0030*	1/2
472,203	472,218	7.74 × 10^−6^	1	TGTATCTTGAAAAACA	−26	*TTHA0507*	S
472,203	472,218	7.74 × 10^−6^	1	TGTTTTTCAAGATACA	−56	*TTHA0506*	S
130,005	130,020	1.01 × 10^−5^	1	TTTATTCTCCCTTACA	−10	*TTHA0133*	1/2
130,005	130,020	1.01 × 10^−5^	1	TGTAAGGGAGAATAAA	−3	*TTHA0132*	S
1506	1521	1.23 × 10^−5^	1	AGTGAGATAACTCACA	−666	*TTHC003*	1/3
1506	1521	1.23 × 10^−5^	1	TGTGAGTTATCTCACT	+627	*TTHC002*	S
79,627	79,642	1.30 × 10^−5^	1	TGTGGTCCAGGCTACC	−78	*TTHB089*	1/3
79,627	79,642	1.30 × 10^−5^	1	GGTAGCCTGGACCACA	−162	*TTHB088*	S
615,132	615,147	1.46 × 10^−5^	1	GGTAGCCAGGGATACA	+909	*TTHA0647*	4/4
1,715,061	1,715,076	1.65 × 10^−5^	1	TGTAGGCCAGGCCACG	−33	*TTHA1833*	1/2
609,145	609,160	1.83 × 10^−5^	1	CGTGTCCCTGAACACA	+790	*TTHA0641*	2/4
614,143	614,158	2.12 × 10^−5^	1	TGTGCCTTTGGCCACA	+326	*TTHA0645*	1/3
1,794,923	1,794,938	2.33 × 10^−5^	1	GGTATGCTCAAGTACA	+13	*TTHA1912*	1/2
1,794,923	1,794,938	2.33 × 10^−5^	1	TGTACTTGAGCATACC	−19	*TTHA1911*	1/4
1272	1287	2.61 × 10^−5^	1	TGTAGCCCAGGCCAAA	+239	*TTHB003*	S
1272	1287	2.61 × 10^−5^	1	TTTGGCCTGGGCTACA	+536	*TTHB004*	4/4
199,120	199,135	2.90 × 10^−5^	1	TGTGGCGTATAACAAA	−17	*TTHA0202*	S
199,120	199,135	2.90 × 10^−5^	1	TTTGTTATACGCCACA	−103	*TTHA0201*	S
357,035	357,050	3.43 × 10^−5^	1	AGTGATGTAAACTAAA	−26	*TTHA0374*	S
314,103	314,118	3.67 × 10^−5^	1	TGTGTTGCAGGACCCA	+58	*TTHA0326*	2/11
1,540,358	1,540,373	3.95 × 10^−5^	1	TGTAGCTTCCCATACC	−67	*TTHA1627*	S
1,540,358	1,540,373	3.95 × 10^−5^	1	GGTATGGGAAGCTACA	+13	*TTHA1626*	S

(*p*-Value) The probability of a random sequence of the same length matching that position of the sequence with an as good or better score. (*Q*-value) False discovery rate if the occurrence is accepted as significant. (Loc) Location of the TTHB099-binding site relative to the start site of transcription. (Gene) Proximal gene downstream of TTHB099 consensus sequence. (Op) Gene position within the postulated operon. (S) No operon, single transcriptional unit.

**Table 3:** Binding kinetics parameters of TTHB099 to potential gene promoter elements.

Gene	Sequence	*k*_on_ (M^−1^s^−1^)	*k*_off_ (s^−1^)	K_D_ (M)	R^2^
*TTHA0080/81*	TGTGTTTTAGTTTACT	122,852	1.145 × 10^−2^	9.322 × 10^−8^	0.9817
*TTHA0506/07*	TGTTTTTCAAGATACA	164,971	1.280 × 10^−2^	7.762 × 10^−8^	0.9718
*TTHA0132/33*	TGTAAGGGAGAATAAA	96,736	2.140 × 10^−2^	2.212 × 10^−7^	0.9687
*TTHB088/89*	GGTAGCCTGGACCACA	214,153	7.163 × 10^−4^	3.345 × 10^−9^	0.9805
*TTHA1833*	TGTAGGCCAGGCCACG	332,611	1.013 × 10^−3^	3.046 × 10^−9^	0.9757
*TTHA1911/12*	TGTACTTGAGCATACC	136,294	8.938 × 10^−3^	6.558 × 10^−8^	0.9806
*TTHA0201/02*	TTTGTTATACGCCACA	57,231	4.464 × 10^−2^	7.801 × 10^−7^	0.9596
*TTHA0374*	AGTGATGTAAACTAAA	−	−	−	−
*TTHA1626/27*	GGTATGGGAAGCTACA	126,605	1.291 × 10^−2^	1.020 × 10^−7^	0.9759

(*TTHA0080/81*) A common TTHB099-binding site shared by two bidirectional promoters. (−) No apparent binding.

**Table 4:** Expression profile data of the FIMO identified operons in a TTHB099-deficient strain of *T. thermophilus* HB8.

Operon	Gene	Role	LogFC	Adj. *p*-Value
S	*TTHA0080*	hypothetical protein	0.851	0.0268
1	*TTHA0081*	hypothetical protein	−0.202	0.421
2	*TTHA0082*	phosphoesterase	−0.176	0.463
3	*TTHA0083*	dimethyladenosine transferase	−0.219	0.336
S	*TTHA0506*	malate synthase	−0.454	0.0983
S	*TTHA0507*	IclR family transcriptional regulator, acetate operon repressor	0.276	0.619
S	*TTHA0132*	hypothetical protein	0.872	0.0295
1	*TTHA0133*	Short-chain dehydrogenase/reductase family oxidoreductase	−0.211	0.674
2	*TTHA0134*	NrdR family transcriptional regulator	−0.328	0.350
S	*TTHB088*	Zn-dependent hydrolase	−0.386	0.653
1	*TTHB089*	hypothetical protein	−0.779	0.0451
2	*TTHB090*	hypothetical protein	−0.0653	0.955
3	*TTHB091*	hypothetical protein	−0.217	0.674
1	*TTHA1833*	ABC transporter permease	−0.294	0.287
2	*TTHA1834*	ABC transporter ATP-binding protein	−0.195	0.567
1	*TTHA1911*	3-isopropylmalate dehydratase large subunit	−0.817	0.0246
2	*TTHA1910*	homoaconitate hydratase small subunit	−1.14	0.0265
3	*TTHA1909*	hypothetical protein	−0.0793	0.790
4	*TTHA1908*	hypothetical protein	−0.0327	0.905
1	*TTHA1912*	hypothetical protein	0.353	0.154
2	*TTHA1913*	hypothetical protein	0.723	0.0284
S	*TTHA0201*	Mg^2+^ chelatase family protein	0.141	0.698
S	*TTHA0202*	hypothetical protein	0.454	0.0644
S	*TTHA0374*	hypothetical protein	0.687	0.0421
S	*TTHA1626*	hypothetical protein	2.62	2.10 × 10^−3^
S	*TTHA1627*	hypothetical protein	−1.20	0.0960

(Operon) Numbers indicate positions of the genes within the operon. (S) Single transcriptional unit. (Role) The biological function identified using the KEGG database [19]. (LogFC) Log2-fold change between data obtained from TTHB099-deficient (accessions GSM530118/20/22) and wild-type (accessions GSM532194/5/6) *T. thermophilus* HB8 strains, SuperSeries GSE21875. (Adj. *p*-value) The *p*-value obtained following multiple testing corrections using the default Benjamini and Hochberg false discovery rate method [25].

**Table 5:** GEO2R analysis of the most affected genes in the absence of TTHB099.

Operon	Gene	Role	LogFC	Adj. *p*-Value
1	*TTHA1498*	Elongation Factor G	+4.384	2.07 × 10^−4^
2	*TTHA1499*	MoxR-like protein	+5.067	7.03 × 10^−5^
3	*TTHA1500*	Phosphoenolpyruvate Synthase	+5.231	7.03 × 10^−5^
4	*TTHA1501*	Hemolysin III	+3.133	1.27 × 10^−3^
5	*TTHA1502*	Response Regulator_two-component system, OmpR family	+1.087	9.51 × 10^−3^
6	*TTHA1503*	Sensor Histidine Kinase	+0.369	2.46 × 10^−1^
S	*TTHA1836*	Isocitrate lyase	+4.423	1.52 × 10^−4^
1	*TTHA1838*	SufC protein, ATP-binding protein	−2.465	1.06 × 10^−3^
2	*TTHA1839*	SufB protein, membrane protein	−2.593	9.53 × 10^−4^
3	*TTHA1840*	SufD protein, membrane protein	−2.630	6.25 × 10^−4^
4	*TTHA1841*	Dioxygenase ferredoxin subunit	−2.419	2.59 × 10^−3^
1	*TTHA1133*	ba3-type cytochrome C oxidase polypeptide IIA	+1.311	4.37 × 10^−2^
2	*TTHA1134*	ba3-type cytochrome C oxidase polypeptide II	+2.944	7.89 × 10^−3^
3	*TTHA1135*	ba3-type cytochrome C oxidase polypeptide I	+4.269	1.27 × 10^−3^
1	*TTHA1136*	hypothetical protein	+1.910	1.29 × 10^−3^
2	*TTHA1137*	Major facilitator superfamily transporter	+2.300	9.53 × 10^−4^
1	*TTHA0251*	Elongation factor Tu	−1.254	1.17 × 10^−2^
1	*TTHA0250*	50S ribosomal protein L33	−1.139	8.04 × 10^−3^
2	*TTHA0249*	Preprotein translocase subunit SecE	−0.997	9.18 × 10^−3^
3	*TTHA0248*	Transcription antitermination protein NusG	−1.136	7.86 × 10^−3^
1	*TTHA0247*	50S ribosomal protein L11	−2.378	1.27 × 10^−3^
2	*TTHA0246*	50S ribosomal protein L1	−1.776	2.16 × 10^−3^
1	*TTHA0084*	NADH-quinone oxidoreductase subunit 7	+1.083	8.73 × 10^−3^
2	*TTHA0085*	NADH dehydrogenase subunit B	+1.005	2.41 × 10^−2^
3	*TTHA0086*	NADH-quinone oxidoreductase subunit 5	+1.251	1.06 × 10^−2^
4	*TTHA0087*	NADH-quinone oxidoreductase subunit 4	+1.255	6.43 × 10^−3^
5	*TTHA0088*	NADH-quinone oxidoreductase subunit 2	+0.693	4.43 × 10^−2^
6	*TTHA0089*	NADH-quinone oxidoreductase subunit 1	+1.249	4.68 × 10^−3^
7	*TTHA0090*	NADH-quinone oxidoreductase subunit 3	+1.248	5.76 × 10^−3^
8	*TTHA0091*	NADH-quinone oxidoreductase subunit 8	+1.490	3.62 × 10^−3^
9	*TTHA0092*	NADH-quinone oxidoreductase subunit 9	+1.502	2.21 × 10^−3^
10	*TTHA0093*	NADH-quinone oxidoreductase subunit 10	+1.626	6.84 × 10^−3^
11	*TTHA0094*	NADH-quinone oxidoreductase subunit 11	+1.043	6.39 × 10^−3^
12	*TTHA0095*	NADH-quinone oxidoreductase subunit 12	+1.492	2.85 × 10^−3^
13	*TTHA0096*	NADH-quinone oxidoreductase subunit 13	+1.679	3.34 × 10^−3^
14	*TTHA0097*	NADH-quinone oxidoreductase subunit 14	+1.509	2.84 × 10^−3^
15	*TTHA0098*	arginyl-tRNA synthetase	+0.397	8.43 × 10^−2^
16	*TTHA0099*	serine protease	+0.106	6.09 × 10^−1^
17	*TTHA0100*	UDP-N-acetylmuramoylalanyl-D-glutamate--2,6-diaminopimelate ligase	+0.520	5.11 × 10^−2^
S	*TTHA1626*	hypothetical protein	+2.616	2.10 × 10^−3^
S	*TTHA1625*	Osmotically inducible protein OsmC	+1.206	3.65 × 10^−3^
1	*TTHA1628*	Iron ABC transporter substrate-binding protein	−2.947	1.83 × 10^−3^
2	*TTHA1629*	Iron ABC transporter permease	−2.344	1.68 × 10^−3^
3	*TTHA1630*	Iron ABC transporter ATP-binding protein	−0.796	1.69 × 10^−2^
4	*TTHA1631*	tRNA pseudouridine synthase A	−0.461	8.43 × 10^−2^
S	*TTHA0135*	MutT/nudix family protein	−1.369	6.82 × 10^−3^
1	*TTHA0206*	nicotinamide nucleotide transhydrogenase subunit alpha 1	+1.516	5.30 × 10^−3^
2	*TTHA0207*	nicotinamide nucleotide transhydrogenase subunit alpha 2	+1.596	2.85 × 10^−3^
3	*TTHA0208*	nicotinamide nucleotide transhydrogenase subunit beta	+1.647	2.10 × 10^−3^
1	*TTHA0209*	50S ribosomal protein L10	−1.673	5.33 × 10^−3^
2	*TTHA0210*	50S ribosomal protein L7/L12	−1.326	8.74 × 10^−3^
1	*TTHB117*	putative type IV pilin	+1.125	4.09 × 10^−2^
2	*TTHB118*	secretion system protein	+1.450	3.74 × 10^−3^
3	*TTHB119*	prepilin-like protein	+1.429	5.85 × 10^−3^
4	*TTHB120*	hypothetical protein	+2.250	1.27 × 10^−3^
1	*TTHA1652*	maltose ABC transporter substrate-binding protein	+1.787	1.72 × 10^−3^
2	*TTHA1651*	maltose ABC transporter permease	+2.154	1.17 × 10^−3^
3	*TTHA1650*	maltose ABC transporter permease	+2.108	1.29 × 10^−3^
1	*TTHB186*	putative transcriptional regulator	+3.377	2.59 × 10^−3^
2	*TTHB187*	hypothetical protein	+2.036	7.58 × 10^−3^
1	*TTHB188*	hypothetical protein	+1.215	9.19 × 10^−3^
2	*TTHB189*	CRISPR-associated Cse2 family protein	+1.514	4.80 × 10^−3^
3	*TTHB190*	hypothetical protein	+1.671	6.62 × 10^−3^
4	*TTHB191*	hypothetical protein	+1.480	4.34 × 10^−3^
5	*TTHB192*	hypothetical protein	+1.669	4.68 × 10^−3^
6	*TTHB193*	hypothetical protein	+1.446	6.84 × 10 ^−3^
7	*TTHB194*	hypothetical protein	+1.549	1.71 × 10^−2^

(Operon) Numbers indicate positions of the genes within the operon. (S) Single transcriptional unit. (Role) The biological function identified using the KEGG database (LogFC) Log2-fold change between data obtained from TTHB099-deficient and wild-type *T. thermophilus* HB8 strains. (Adj. *p*-value) The *p*-value obtained following multiple testing corrections using the default Benjamini and Hochberg false discovery rate method [25].

## Supplementary Materials

The following are available online at https://www.mdpi.com/1422-0067/21/21/7929/s1, Table S1: EMSA quantification data, Table S2: GEO2R analysis of expression profiles from TTHB099-deficient and wild type *T. thermophilus* HB8 strains, Table S3: Oligonucleotides, Figure S1: Quantitative densitometry analysis of TTHB099 binding to its palindromic consensus sequence, Figure S2: Expression, purification, and quantification of TTHB099 protein, Data S1: REPSA Round 7 fastq sequences, and Data S2: REPSA Round 7 Sequencing1-processed sequences can be found at https://www.mdpi.com/1422-0067/21/21/7929/s1.

## Abbreviations

BLI	Biolayer Interferometry
cAMP	3′,5′-cyclic Adenosine Monophosphate
CRP	Cyclic AMP Receptor Protein
DOOR^2^	Database of PrOkaryotic OpeRons
EMSA	Electrophoretic Mobility Shift Assay
ETC	Electron Transport Chain
FIMO	Find Individual Motif Occurrences
GEO	Gene Expression Omnibus
IISRE	Type IIS Restriction Endonuclease
IRD7	IRDye^®^ 700
ISP	Individual Sequencing Particle
KEGG	Kyoto Encyclopedia of Genes and Genomes
MEME	Multiple Em for Motif Elicitation
NCBI	National Center for Biotechnology Information
PGM	Personal Genome Machine
REPA	Restriction Endonuclease Protection Assay
REPSA	Restriction Endonuclease Protection, Selection, and Amplification
TetR	Tetracycline Repressor Protein
TF	Transcription Factor
TFBS	Transcription Factor Binding Site
TSS	Transcription Start Site
