# Supplementary material for: Determination and Dissection of DNA-Binding Specificity for the Thermus thermophilus HB8 Transcriptional Regulator TTHB099
Source: Int J Mol Sci. 2020 Oct 26;21(21):7929. doi: 10.3390/ijms21217929 (PMC7662524; doi:10.3390/ijms21217929)
Supplement: Supplementary file 1 [file ijms-21-07929-s001.zip › Figure S1.pdf]

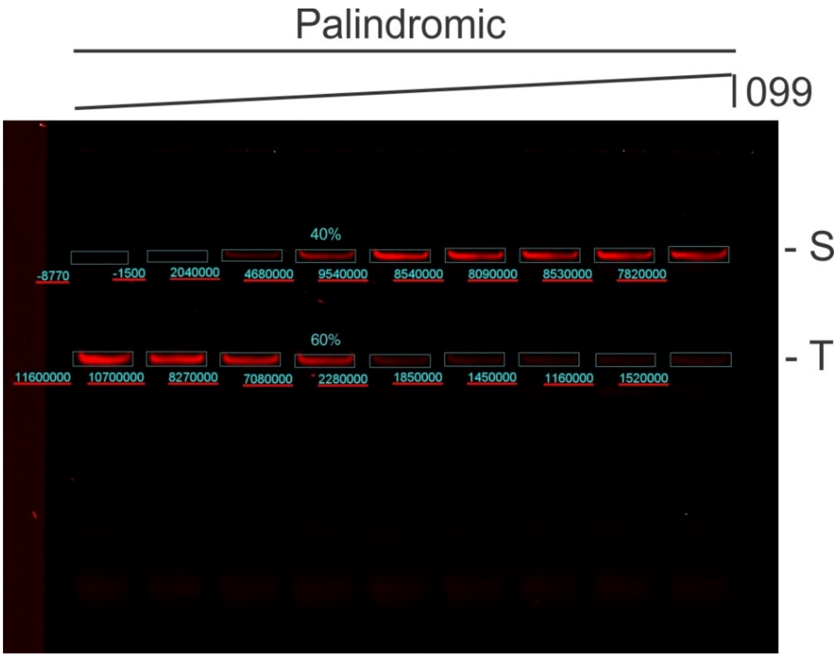

Figure S1. Quantitative densitometry analysis of TTHB099 binding to its palindromic consensus sequence. Shown is an IR fluorescence image of IRD700-labeled ST2_099 incubated with 0, 0, 0.66, 1.32, 2.64, 5.27, 10.5, 21.1, or 42.2 nM TTHB099 protein. K_D is determined as 1.9 nM using data at the binding midpoint (2.64 nM TTHB099). (S) Protein-DNA complex, and (T) uncomplexed DNA.
