# Supplementary material for: Determination and Dissection of DNA-Binding Specificity for the Thermus thermophilus HB8 Transcriptional Regulator TTHB099
Source: Int J Mol Sci. 2020 Oct 26;21(21):7929. doi: 10.3390/ijms21217929 (PMC7662524; doi:10.3390/ijms21217929)
Supplement: Supplementary file 1 [file ijms-21-07929-s001.zip › Figure S2.pdf]

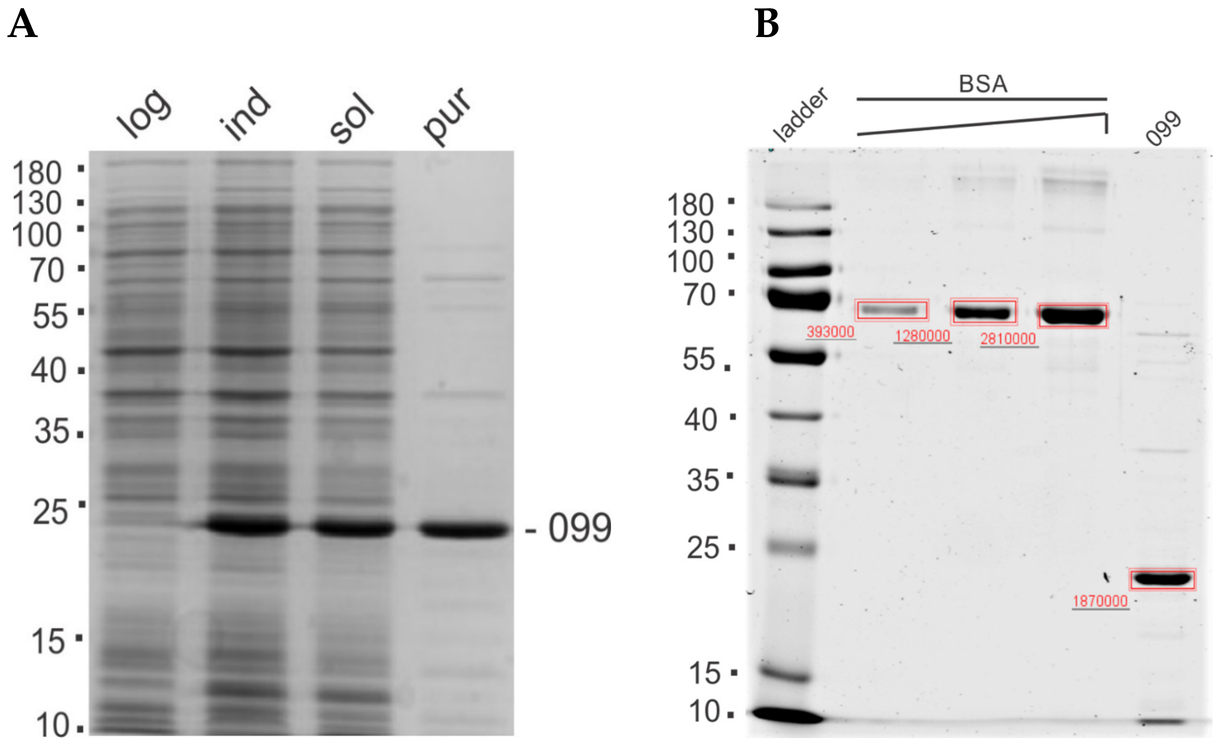

Figure S2. Expression, purification, and quantification of TTHB099 protein. (**A**) Shown is a Coomassie Blue G-250 stained 12% SDS-PAGE gel onto which was loaded whole cell extracts or partially purified fractions equivalent to 0.2% of the total preparation. Lanes shown left to right: (log) logarithmic growth bacteria, (ind) bacteria following IPTG-induction for 4 h, (sol) soluble proteins following sonication and centrifugation, (pur) 2.3 μg purified TTHB099 protein. The location of molecular weight standards are indicated at the left of the figure. (**B**) Quantitative densitometric analysis of a Coomassie Blue G-250 stained 12% SDS-PAGE gel containing a BSA standard curve (left to right: 0.5, 1, 2 mg protein) and 0.5 μL stock TTHB099. The final concentration of TTHB099 is estimated to be 50.6 μM.
