# Supplementary material for: Determination and Dissection of DNA-Binding Specificity for the Thermus thermophilus HB8 Transcriptional Regulator TTHB099
Source: Int J Mol Sci. 2020 Oct 26;21(21):7929. doi: 10.3390/ijms21217929 (PMC7662524; doi:10.3390/ijms21217929)
Supplement: Supplementary file 1 [file ijms-21-07929-s001.zip › Table S1.pdf]

Table S1. EMSA quantification data.

Lane	TTHB099 (nM)	Intensity S	Intensity T
1	0	–8770	11,600,000
2	0	–1500	10,700,000
3	0.66	2,040,000	8,270,000
4	1.32	4,680,000	7,080,000
5	2.64	9,540,000	2,280,000
6	5.27	8,540,000	1,850,000
7	10.5	8,090,000	1,450,000
8	21.1	8,530,000	1,160,000
9	42.2	7,820,000	1,520,000

Quantitation calculations of densitometric data from Figure S2 were performed as previously described [9]. (TTHB099) Final TTHB099 concentration in binding reaction. (Intensity S) Pixel number for TTHB099-DNA species. (Intensity T) Pixel number for unbound DNA.
