# Supplementary material for: Determination and Dissection of DNA-Binding Specificity for the Thermus thermophilus HB8 Transcriptional Regulator TTHB099
Source: Int J Mol Sci. 2020 Oct 26;21(21):7929. doi: 10.3390/ijms21217929 (PMC7662524; doi:10.3390/ijms21217929)
Supplement: Supplementary file 1 [file ijms-21-07929-s001.zip › Table S3.pdf]

Table S3. Oligonucleotides

Name	Sequence	Length	Purif.	Use
ST2R24	CTAGGAATTCGTGCAGAGGTGAATNNNNNNNNNNNNNNNNNNNNNNNNTTACCATCCCTCCAGAAGCTTGGAC	73	PAGE	ST2R24 Template Precursor
ST2L	CTAGGAATTCGTGCAGAGGTGAAT	24	Desalt	PCR Left Primer
ST2Ls	CTAGGAATTCGTGCAGAGGTGA	22	Desalt	PCR Left Primer Short
ST2R	GTCCAAGCTTCTGGAGGGATGGTAA	25	Desalt	PCR Right Primer
IRD7_ST2R	/5IRD700/GTCCAAGCTTCTGGAGGGATGGTAA	25	HPLC	5′-IRDye700 PCR Primer
ABC01_ST2R	CCATCTCATCCCTGCGTGTCTCCGACTCAGCTGCAAGTTCGATGTCCAAGCTTCTGGAGGGATG	64	PAGE	Fusion PCR Primer
trP1_ST2L	CCTCTCTATGGGCAGTCGGTGATCTAGGAATTCGTGCAGAGGTGA	45	PAGE	Fusion PCR Primer
A_uni	CCATCTCATCCCTGCGTG	18	Desalt	PCR Primer
trP1_uni	CCTCTCTATGGGCAGTCGG	19	Desalt	PCR primer
IRD8_trP1_ST2L	/5IRD800/CCTCTCTATGGGCAGTCGGTGATCTAG	27	HPLC	5′-IRDye800- modified PCR Primer
Bio_ST2R	/5BiodT/GTCCAAGCTTCTGGAGGGATG	22	HPLC	5′-biotinylated PCR primer
REPSAis	CTAGGAATTCGTGCAGAGGTGAATCGTCATAGAATTCGTTACCATCCCTCCAGAAGCTTGGAC	63	PAGE	REPSAis control DNA precursor
ST2_099_wt	AGGAATTCGTGCAGAGGTGAATTGTATTCTAGAATACATTACCATCCCTCCAGAAGCTTG	65	Desalt	TTHB099 consensus probe
ST2_099_wt_m1	AGGAATTCGTGCAGAGGTGAATGGTATTCTAGAATACATTACCATCCCTCCAGAAGCTTG	65	Desalt	TTHB099 mutant 1 probe precursor
ST2_099_wt_m2	AGGAATTCGTGCAGAGGTGAATTTTATTCTAGAATACATTACCATCCCTCCAGAAGCTTG	65	Desalt	TTHB099 mutant 2 probe precursor

ST2_099_wt_m3	AGGAATTCGTGCAGAGGTGAATTGAATTCTAGAATACATTACCATCCCTCCAGAAGCTTG	65	Desalt	TTHB099 mutant 3 probe precursor
ST2_099_wt_m4	AGGAATTCGTGCAGAGGTGAATTGTCTTCTAGAATACATTACCATCCCTCCAGAAGCTTG	65	Desalt	TTHB099 mutant 4 probe precursor
ST2_099_wt_m5	AGGAATTCGTGCAGAGGTGAATTGTACTCTAGAATACATTACCATCCCTCCAGAAGCTTG	65	Desalt	TTHB099 mutant 5 probe precursor
ST2_099_wt_m6	AGGAATTCGTGCAGAGGTGAATTGTATACTAGAATACATTACCATCCCTCCAGAAGCTTG	65	Desalt	TTHB099 mutant 6 probe precursor
ST2_099_wt_m7	AGGAATTCGTGCAGAGGTGAATTGTATTTTAGAATACATTACCATCCCTCCAGAAGCTTG	65	Desalt	TTHB099 mutant 7 probe precursor
ST2_099_wt_m8	AGGAATTCGTGCAGAGGTGAATTGTATTCAAGAATACATTACCATCCCTCCAGAAGCTTG	65	Desalt	TTHB099 mutant 8 probe precursor
ST2_099_0080(0081) p	AGGAATTCGTGCAGAGGTGAATTGTGTTTTAGTTTACTTTACCATCCCTCCAGAAGCTTG	60	Desalt	*TTHA0080(0081)* promoter DNA probe precursor
ST2_099_0030p	AGGAATTCGTGCAGAGGTGAATTGTGTACGAAATTACATTACCATCCCTCCAGAAGCTTG	60	Desalt	*TTHA0030* promoter DNA probe precursor
ST2_099_0506(0507) p	AGGAATTCGTGCAGAGGTGAATTGTTTTTCAAGATACATTACCATCCCTCCAGAAGCTTG	60	Desalt	*TTHA0506(0507)* promoter DNA probe precursor
ST2_099_0132(0133) p	AGGAATTCGTGCAGAGGTGAATTGTAAGGGAGAATAAATTACCATCCCTCCAGAAGCTTG	60	Desalt	*TTHA0132(0133)* promoter DNA probe precursor
ST2_099_C002(C003) p	AGGAATTCGTGCAGAGGTGAATTGTGAGTTATCTCACTTTACCATCCCTCCAGAAGCTTG	60	Desalt	*TTHC002(C003)* promoter DNA probe precursor
ST2_099_B088(B089) p	AGGAATTCGTGCAGAGGTGAATGGTAGCCTGGACCACATTACCATCCCTCCAGAAGCTTG	60	Desalt	*TTHB088(B089)* promoter DNA probe precursor
ST2_099_0647p	AGGAATTCGTGCAGAGGTGAATGGTAGCCAGGGATACATTACCATCCCTCCAGAAGCTTG	60	Desalt	*TTHA0647* promoter DNA probe precursor

ST2_099_1833p	AGGAATTCGTGCAGAGGTGAATTGTAGGCCAGGCCACGTTACCATCCCTCCAGAAGCTTG	60	Desalt	*TTHA1833* promoter DNA probe precursor
ST2_099_0641p	AGGAATTCGTGCAGAGGTGAATCGTGTCCCTGAACACATTACCATCCCTCCAGAAGCTTG	60	Desalt	*TTHA0641* promoter DNA probe precursor
ST2_099_0645p	AGGAATTCGTGCAGAGGTGAATTGTGCCTTTGGCCACATTACCATCCCTCCAGAAGCTTG	60	Desalt	*TTHA0645* promoter DNA probe precursor
ST2_099_1911(1912) p	AGGAATTCGTGCAGAGGTGAATTGTACTTGAGCATACCTTACCATCCCTCCAGAAGCTTG	60	Desalt	*TTHA1911(1912)* promoter DNA probe precursor
ST2_099_B003(B004) p	AGGAATTCGTGCAGAGGTGAATTGTAGCCCAGGCCAAATTACCATCCCTCCAGAAGCTTG	60	Desalt	*TTHB003(B004)* promoter DNA probe precursor
ST2_099_0201(0202) p	AGGAATTCGTGCAGAGGTGAATTTTGTTATACGCCACATTACCATCCCTCCAGAAGCTTG	60	Desalt	*TTHA0201(0202)* promoter DNA probe precursor
ST2_099_0374p	AGGAATTCGTGCAGAGGTGAATAGTGATGTAAACTAAATTACCATCCCTCCAGAAGCTTG	60	Desalt	*TTHA0374* promoter DNA probe precursor
ST2_099_0326p	AGGAATTCGTGCAGAGGTGAATTGTGTTGCAGGACCCATTACCATCCCTCCAGAAGCTTG	60	Desalt	*TTHA0326* promoter DNA probe precursor
ST2_099_1626(1627) p	AGGAATTCGTGCAGAGGTGAATGGTATGGGAAGCTACATTACCATCCCTCCAGAAGCTTG	60	Desalt	*TTHA1626(1627)* promoter DNA probe precursor
